# IMP-1 encoded by a novel Tn*402*-like class 1 integron in clinical *Achromobacter xylosoxidans*, China

**DOI:** 10.1038/srep07212

**Published:** 2014-11-27

**Authors:** Zhenhong Chen, Haihong Fang, Li Wang, Fengjun Sun, Yong Wang, Zhe Yin, Huiying Yang, Wenhui Yang, Jie Wang, Peiyuan Xia, Dongsheng Zhou, Changting Liu

**Affiliations:** 1 Nanlou Respiratory Diseases Department, Chinese People's Liberation Army General Hospital, Beijing 100853, China; 2 State Key Laboratory of Pathogen and Biosecurity, Beijing Institute of Microbiology and Epidemiology, Beijing 100071, China; 3 Department of Pharmacy, Southwest Hospital, the Third Military Medical University, Chongqing 400038, China; 4 Institute of Disease Control and Prevention, Beijing 100071, China

## Abstract

*Achromobacter xylosoxidans* strain A22732 is isolated from a pneumonia patient in China and produces carbapenemases OXA-114e and IMP-1, which are encoded by chromosome and plasmid, respectively, and confer resistance to multiple ß-lactam antibiotics including carbapenems. The *bla*_IMP-1_ gene together with *aacA7* and *orfE* is captured by a novel Tn*402*-like class 1 integron in a conjugative IncP-1ß plasmid. In addition to the intrinsic integron promoter PcW, there is still a *bla*_IMP-1_ gene cassette-specific promoter. This is the first report of carbapenemase-encoding IncP-1ß plasmid in clinical bacterial isolate.

A*chromobacter xylosoxidans* is a Gram-negative, motile bacterium from the *Achromobacter* genus of the *Alcaligenaceae* family, and frequently found in water environments and can also cause opportunistic infections especially in immunocompromised patients.

*A. xylosoxidans* expresses a chromosomally encoded intrinsic carbapenemase OXA-114 (Ambler class D) in a constitutive manner^1^ with at least 22 variants OXA-114a to OXA-114v (http://www.ncbi.nlm.nih.gov/nuccore/, last accessed May 9, 2014), and PCR detection of *bla*_OXA-114_ genes has been established for species identification of this bacterium^2^.

The IMP-type metallo-carbapenemases (class B) are composed of at least 48 variants IMP-1 to IMP-48 (http://www.lahey.org/studies/, last accessed May 9, 2014), and they are found in many clinical Gram-negative bacteria including *Pseudomonas spp.*, *Acinetobacter spp.*, and members of the *Enterobacteriaceae* family^3^. At least three IMP variants, namely IMP-1 and IMP-19^4^, and IMP-10^5^, have been detected in *A. xylosoxidans* isolates from Japan; all of these *bla*_IMP-1_ genes are carried on plasmid-borne class 1 integrons, but the complete nucleotide sequences of these IMP-encoding plasmids are not determined.

The present study describes the complete nucleotide sequence of a conjugative IncP-1ß plasmid from a clinical *A. xylosoxidans* isolate from China, carrying a novel Tn*402*-like class 1 integron that includes the *bla*_IMP-1_ gene cassette.

## Results and Discussion

### *A. xylosoxidans* strain A22732 harboring a conjugative IMP-encoding plasmid

In September 2010, an 86-year-old male was admitted to our hospital and diagnosed to have pneumonia, and sputum specimens were sampled on the same day. The next day, bacterial growth was observed after cultivation of sputum on Mueller-Hinton agar, and the bacterial isolate designated A22732 was identified as *A. xylosoxidans* by VITEK 2, Bruker MALDI Biotyper, and 16s rRNA gene sequencing. The antimicrobial susceptibility test using VITEK 2 indicated A22732 was resistant to multiple β-lactam antibiotics including imipenem and meropenem but remained susceptible to fluoroquinolones, and the patient then received intravenous administration with moxifloxacin hydrochloride, and he was cured after ten days of antimicrobial treatment.

Positive PCR amplification of two carbapenemase genes *bla*_IMP_ and *bla*_OXA-114_ was observed for strain A22732, which was further validated by PCR amplicon sequencing, but all the remaining known carbapenemase and extended spectrum ß-lactamase (ESBL) genes tested gave negative PCR results. The whole gene amplification/sequencing indicated the presence of intact *bla*_OXA-114e_ gene in A22732. A *bla*_IMP_-positive and *bla*_OXA-114_-negative *E. coli* transconjugant, designated A22732-IMP-EC600, was obtained by conjugal transfer, indicating that A22732 harbored a conjugative IMP-encoding plasmid, which was designated pA22732-IMP. As determined by a modified CarbaNP test^6^, strain 22732-IMP-EC600 had class B carbapenemase activity, while A22732 probably expressed class B/D carbapenemases (Fig S1), being consistent with the above PCR/sequencing results.

The minimum inhibitory concentration (MIC) values (Table 1) were determined for A22732, 22732-IMP-EC600, and EC600. A22732 and 22732-IMP-EC600 show almost identical drug resistance profiles. These two strains are highly resistant to penicillins, aztreonam, and ephalosporins tested, but they remain susceptible to the fluoroquinolone, furane, aminoglycoside, and sulfanilamide drugs tested. Both A22732 and 22732-IMP-EC600 are resistant to imipenem and meropenem, but the MIC values of imipenem and meropenem (both > = 16) against A22732 are much higher than those (4 and 2, respectively) against 22732-IMP-EC600, which is consistent with the fact that A22732 expresses two different arbapenemase IMP-1 and OXA-114e while 22732-IMP-EC600 produces only a single one IMP-1.

### IncP-1ß plasmid pA22732-IMP

**Figure 1:**
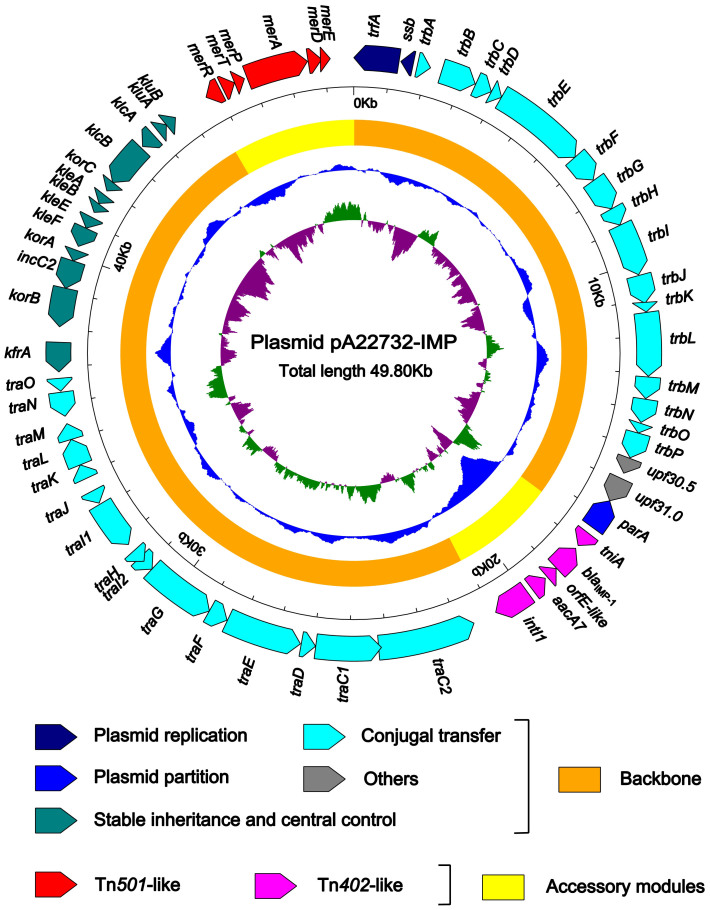
Schematic maps of plasmid pA22732-IMP. Genes are denoted by arrows and colored based on gene function classification. The innermost circle presents GC-Skew [(G-C)/(G+C)] with a window size of 500-bp and a step size of 20-bp. The blue circle presents GC content. Shown also are backbone (orange) and accessory module (yellow) regions of pA22732-IMP.

The complete nucleotide sequence of pA22732-IMP was determined by a next-generation sequencing approach with a 160-fold coverage. pA22732-IMP has a circular DNA sequence of 49,804-bp with a total of 60 complete genes annotated and an average G+C content of 65.07 (Fig 1). The backbone of pA22732-IMP is composed of DNA regions for plasmid replication (*ssb* and *trfA*), partition (*parA*), stable inheritance and central control (the *ctl* region: *kluB* to *kfrA*), conjugal transfer (*trbA* to *trbP,* and *traO* to *traC*), and unknown functions (*upf30.5* and *upf31.0*) (Fig 1), which show >99% identity to the corresponding regions of a set of IncP-1ß broad-host-range plasmids R751, pB3, pAKD1, pTP6, pADP1, pAMMD1, pB8, pUO1, pAKD15, pAKD17, pB10, pAKD14, pAKD29, pAKD18, pAKD33, pAKD31, and pJP4^7^. The pA22732-IMP backbone of 41,970-bp has a G+C content of 66.04, while the remaining accessory modules have a lower G+C content of 59.85, indicating distinct origins of backbone and accessory elements (Fig 1).

**Figure 2:**
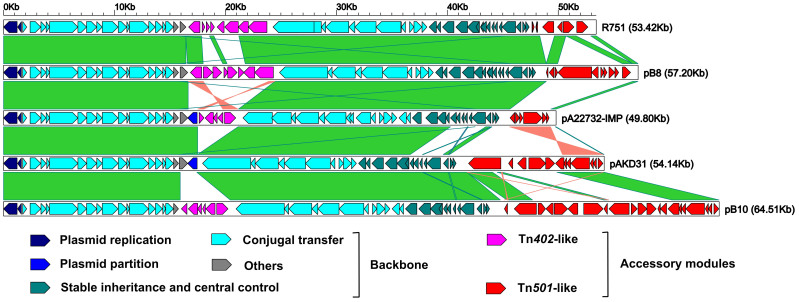
Linear comparisons of sequenced plasmids. Genes are denoted by arrows and colored based on gene function classification.

The entire pA22732-IMP sequence had >70% query cover and >80% identity with the above IncP-1ß plasmids. pA22732-IMP was aligned with four representative plasmids R751 (accession number U67194)^8^, pB10^9^ (AJ564903), pB8 (AJ863570)^10^, and pAKD31 (JQ436721)^11^ through pairwise whole plasmid sequence comparison (Fig 2). R751 is from a clinical *Enterobacter aerogenes* isolate and represents the prototype IncP-1β drug-resistance plasmid, pB10 is isolated from a bacterial community residing in activated sludge compartment of a waste-water treatment plant, and pB8 and pAKD31 are from agricultural soil bacteria. The above IncP-1ß plasmids were selected for further analyses, because they harbored different forms of Tn*501*-like or Tn*40*2-like elements (see below).

The backbone regions are highly conserved in pA22732-IMP, R751, pB10, pB8, and pAKD31. All the former four plasmids harbor two accessory modules, namely Tn*501*-like element and Tn*40*2-like integron, each of which has different gene organizations among these four plasmids (Fig 2). The above two accessory modules are inserted into downstream of *trfA* and that of *traC2*, respectively, which are the targeting locations typically interrupted by mobile elements in IncP-1β plasmids. By contrast, only Tn*501*-like element rather than Tn*40*2-like integron is found in the corresponding region of pAKD31(Fig 2).

### Tn501-like elements

**Figure 3:**
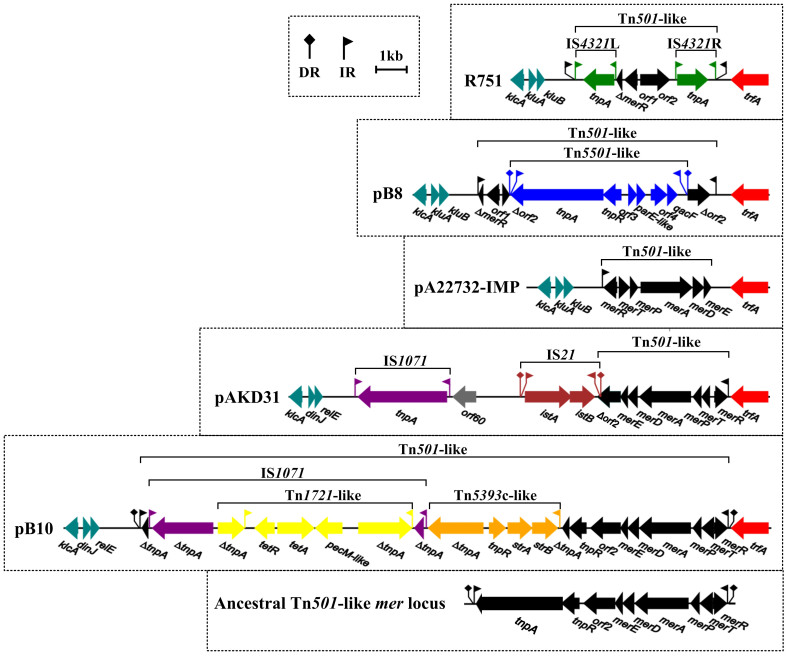
Linear comparisons of Tn*501*-like elements. Genes are denoted by arrows and colored based on gene function classification.

Tn*501*-like transposon insertions, which often harbor the *mer* loci conferring mercuric chloride resistance, are frequently found in IncP-1ß plasmids from agricultural soils^7^,^9^,^10^,^12^. The ancestral Tn*501*-like *mer* locus (Fig 3) contains the Tn*501* transposition genes *tnpA* (transposase) and *tnpR* (resolvase), the mercury-resistance genes *merR* (repressor of *mer* locus), *merT* (integral membrane protein for mercuric transport), *merP* (periplasmic mercury ion-binding protein), *merA* (mercuric reductase), *merD* (co-regulator protein), *merE* (integral membrane protein for mercuric transport) and *orf2* (EAL-domain-containing protein), and the 38-bp terminal inverted repeats (left terminal inverted repeat IRi, and right terminal repeat IRt)^7^.

Tn*501*-like loci can be identified in all the above five plasmids (Fig 3). The Tn*501*-like element of pB10 is flanked by a 5-bp target site (direct repeats of TGCCT), but those of all the other four plasmids leave no trace of insertion.

pB10 harbors an greatly extended Tn*501*-like *mer* locus, because *tnpA* is interrupted by IS*1071* (which is further interrupted by Tn*1721*-like tetracycline-resistance locus) plus a Tn*5393*c-like streptomycin resistance locus, thereby leading to partial deletion of *tnpA*. Loss of IRi and *tnpAR* as well as 3'-terminus deletion of *orf2*, because of insertion of IS*1071* plus IS*21* into *orf2*, is observed for the Tn*501*-like *mer* locus of pAKD31. The Tn*501*-like *mer* locus of pA22732-IMP has undergone loss of IRi, *tnpAR*, and *orf2* as well as an inversion event of the whole locus.

Both R751 and pB8 carry a ‘cryptic’ Tn*501*-like element, which is composed of IRi*, orf1, orf2*, a *merR* remnant and IRt in the absence of all the other features identified for the ancestral Tn*501*-like *mer* locus. In addition, R751 has acquired two copies of IS*4321* (IS*4321*L and IS*4321*R), which flank the *merR* remnant and *orf2*, respectively. By contrast, evolution of pB8 involves insertion of a Tn*501*-like quaternary ammonium compound resistance (*qacF*) locus into *orf2*, thereby disrupting this gene.

### *Tn402-like* integrons

**Figure 4:**
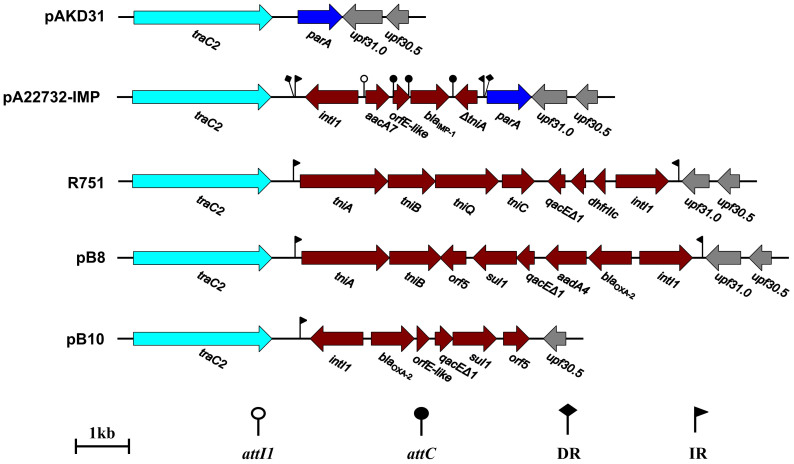
Schematic representation of Tn*402*-like integrons and flanking regions. Genes are denoted by arrows and colored based on gene function classification.

Tn*402* is bound by 25-bp IRi and IRt^10^,^13^, and acts as the primary carrier element of class 1integrons^14^,^15^,^16^,^17^. Tn*402*-like class 1 integrons are indentified in pB8, pA22732-IMP, R751 and pB10, but not pAKD31 (Fig 4a). The pB8 integron is a typical class 1 integron integron that, from 5′ to 3′ side, harbors 5′-conserved segment (CS)-specific integrase gene *intI1*, resistance gene cassettes *bla*_OXA-2_ (class D ß-lactamase OXA-2) and *aadA4* (spectinomycin/streptomycin resistance), 3′-CS-specific gene cluster *qacEΔ1-sul1-orf5*, and Tn*402* transposition genes *tniAB*^10^. The pA22732-IMP integron contains *intI1*, *aacA7* (gentamicin/amikacin resistance), *orfE*, *bla*_IMP-1_, and truncated *tniA*, being atypical due to lack of 3′-CS. Notably, 3′-CS-lacking integrons have been already observed in many cases^16^,^18^,^19^. The R751 integron contains *dhfrIIc* (trimethoprim resistance), *orfD*, truncated *qacE*, and complete Tn*402* transposition module *tniABQC*^13^.The pB10 integron harbors *intI1*, *bla*_OXA-2_, *orfE*, and *qacEΔ1-sul1-orf5*^9^.

At least three key steps (Fig S2) are involved in evolution of Tn*402*-like class 1 integrons^14^,^15^,^16^,^17^: step I, insertion of ancestor class 1 integron (lack of 3′- CS) into Tn*402* (harboring complete *tniABQC* transposition module) to generate a hybrid structure, combining the ability of integron to capture environmental gene cassettes to the mobility of Tn*402* into plasmids and other genetic platforms, which might occur prior to or concomitant with antibiotic era including capture of *qacE* (quaternary ammonium compound resistance); step II, capture of *sul1* (sulfonamide resistance) and *orf5*, and then formation of 3′-CS due to deletion events between *qacE* and *sul1*; step III, deletion events within *tniABQR*, making Tn*402* transposition incompetent.

The pB8 and pB10 integrons, each of which contain 5′-CS, drug resistance gene cassettes, 3′-CS, and completely or partially truncated *tni* module, appear to undergone all the above three steps of evolution. By contrast, the evolution step II (generation of 3′-CS) is mostly likely omitted for the pA22732-IMP integron, while the R751 integron might represent a primitive Tn*402*-like integron due to absence of evolution steps II and III (truncation of *tni* module).

The Tn*402*-like integron of pA22732-IMP is inserted into the *traC*-*parA* intergenic region, leaving *parA* and its downstream gene *upf31.0* intact. The *parA* upstream or around region represents a hot spot for Tn*402* targeting, most likely due to it contains the multimer resolution site II for recognition by Tn*402* transposase^20^,^21^. Interestingly, the insertion of Tn*402*-like integrons into the hotspot target region leads to further deletion of *parA* from pB8 and R751, and that of *parA*/*upf31.0* from pB10.

The pA22732-IMP, R751 and pB8 integrons contains IRi and IRt of Tn*402*, but only IRi rather than IRt is identified for pB10. The lack of IRt in pB10 might due to the above-mentioned deletion removing of *parA*/*upf31.0*. The 5-bp target site (direct repeats of AGCAT) is still intact to flank the pA22732-IMP integron, but all the other three integrons do not leave traces of insertion.

Integrase IntI1 recognizes two different types of recombination site *attI1* (integron attachment site) and *attC* (recognition site for integrase), and it catalyzes integration or excision of gene cassettes through site-specific recombination commonly between one *attI1* site and one or more *attC* sites^22^,^23^. One *attI1* site and three *attC* sites, upstream of *aacA7*, *orfE*, *bla*_IMP-1_ and *tniA*, respectively, are indentified in the pA22732-IMP integron (Fig 4a). These sites are long inverted-repeat-containing sequences of variable length and sequence, and each inverted repeat begins with a core sequence RYYYAAC and ends with an inverted core sequence GTTRRRY as described previously^24^,^25^, which would form imperfect cruciform structures and be required for capture of *aacA7*, *orfE*, and *bla*_IMP-1_.

### Expression of integron gene cassettes

**Figure 5:**
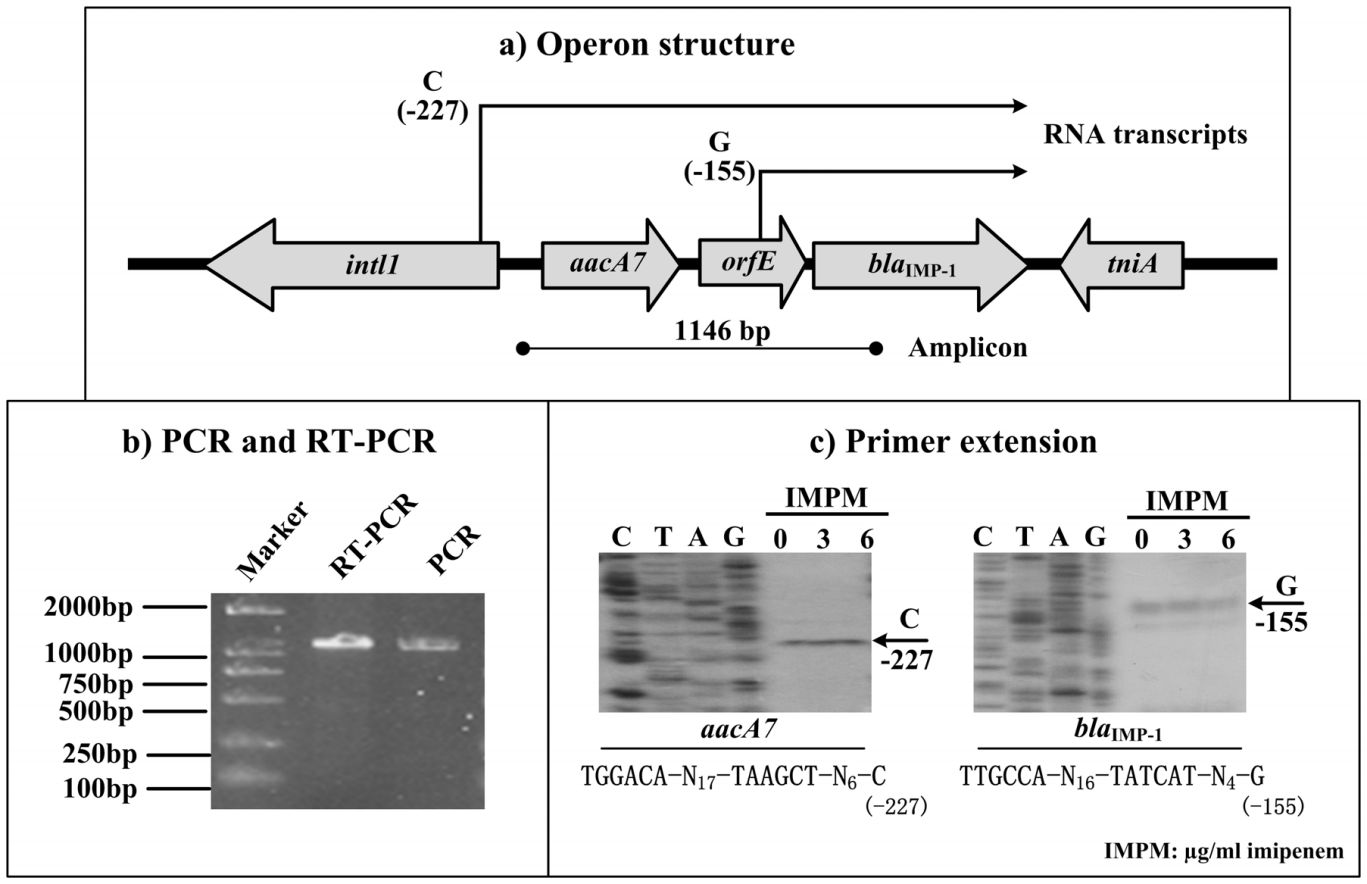
Organization and expression of integron gene cassettes. a) Operon structure. Boxed arrows stand for length/direction of indicated genes. The two broken-line arrows represent primary RNA transcripts transcribed for the *aacA7-orfE-bla*_IMP-1_ operon and the *bla*_IMP-1_ gene, respectively. Line with filled circles at both termini indicates location of primer pair plus expected PCR amplicon. b) PCR and RT-PCR. cDNAs generated from total RNA of strain A22732, and genomic DNA of A22732 were used as templates for RT-PCR and PCR, respectively. c) Primer extension. Primer extension assay of the RNA transcript of *aacA7* or *bla*_IMP-1_ was done for A22732 cultured with addition of increasing amounts of imipenem. Lanes C, T, A and G represent Sanger sequencing reactions. The transcription start of *aacA7* or *bla*_IMP-1_is indicated by the arrow with nucleotide T or G, respectively, and the minus number under arrow indicate the nucleotide position upstream of the *aacA7* or *bla*_IMP-1_ start codon. Representative data from at least two independent biological replicates are shown.

The pA22732-IMP integron gene cassettes *aacA7, orfE* and *bla*_IMP-1_, but not *intI1* and *tniA*, are organized in the same transcriptional direction (Fig 5a). PCR generates an amplicon ranged from 5′-untranslated region (5′-UTR) of *aacA7* to 5′-terminal of the *bla*_IMP-1_ coding region, when using A22732 genomic DNA as template (Fig 5a). The positive RT-PCR amplification with the same primer pair, using cDNA sample generated from A22732 total RNA as template (Fig 5b), indicates that *aacA7*, *orfE*, and *bla*_IMP-1_ are transcribed into a single RNA transcript and thereby, these three genes constitutes a single operon *aacA7-orfE-bla*_IMP-1_ (Fig 5a).

Integrons act as natural gene expression platforms due to the presence of an intrinsic promoter (Pc) that is recognized by RNA polymerase to drive transcription of inserted gene cassettes that generally do not have their own promoters. At least eight distinct types of Pc promoter, PcS (strong, TTGACA-N_17_-TAAACT), PcW (weak, TGGACA-N_17_-TAAGCT), PcH1 (hybrid 1, TGGACA-N_17_-TAAACT), PcH2 (Hybrid 2, TTGACA-N_17_-TAAGCT), PcSS (super-strong, TTGATA-N_17_-TAAACT), PcIn42 (TTGGCA-N_17_-TAAACT), PcIn116 (TTGACA-N_17_-TGAACT), and PcPUO (TCGACA-N_17_-TAAACT) have been described for class 1 integrons^26^. As detected by primer extension (Fig 5c), a transcription start site (nucleotide C) is located at 227-bp upstream of *aacA7* start codon (i.e. a 227-bp 5′-UTR of *aacA7*), validating presence of the PcW promoter to drive *aacA7-orfE-bla*_IMP-1_ transcription. The PcW promoter can be found for all the pA22732-IMP, pB8, R751, and pB8 integrons.

Remarkably, the primer extension assay detects another transcription start site (nucleotide G) which is located at 155-bp upstream of *bla*_IMP-1_ start codon. This assay discloses presence of an internal promoter (TTGCCA-N_16_-TATCAT) driving transcription of the *bla*_IMP-1_ gene cassette (Fig 5c). Being very rare, the internal gene cassette-specific promoter is an extra element in evolution of antimicrobial resistance phenotype and act independent of Pc promoter^27^.

In addition, the primer extension assay shows that addition of increasing amounts of imipenem during bacterial cultivation has no effect on promoter activity of either *aacA7-orfE-bla*_IMP-1_ or *bla*_IMP-1_ (Fig 5c), validating constitutive expression of the *aacA7-orfE-bla*_IMP-1_ operon and the *bla*_IMP-1_ gene cassette. Notably, constitutive transcription of integron gene cassettes has been suggested previously^23^,^28^,^29^.

### Concluding remarks

We present the first complete sequence of IMP-encoding plasmid from *A. xylosoxidans*, and this is also the first report of identification of a carbapenemase-encoding IncP-1ß plasmid from a clinical bacterial isolate. The detected *bla*_IMP-1_ gene is captured by a novel class 1 integron with a novel gene cassette array in the IncP-1ß plasmid pA22732-IMP. The class 1 integron is embedded in a Tn*402*-like transposon and inserted into pA22732-IMP by transposition. Most of the characterized IncP-1ß plasmids are isolated from bacteria in agricultural soils or waters and frequently associated with mercury resistance^7^,^9^,^10^,^11^,^13^. Only a few of them, e.g. pA22732-IMP and R75, are of clinical origins and harbor an array structure of multiple resistance gene cassettes. The IncP-1ß plasmids thus could represent important vehicles for spreading clinically relevant resistance determinants across a number of bacterial species. The transfer of *bla*_IMP_ to *A. xylosoxidans*, which already carried *bla*_OXA-114_ intrinsically, would lead to more severe drug resistance, making high difficulty in timely choosing sensitive antibiotics for treatment. The IMP-producing *A. xylosoxidans* should be taken seriously as the surveillance target especially in East Asia countries such as China and Japan.

## Methods

### Bacteria isolation and identification

Fresh sputum specimens were sampled from the indicated patient and inoculated onto Mueller-Hinton agar for bacterial isolation. The use of human specimens and all related experimental protocols were approved by the Committee on Human Research of Chinese People's Liberation Army General Hospital and carried out in accordance with the approved guidelines, and moreover the informed consent was obtained from the indicated patient. Single colony of each bacterial strain tested was subjective for species identification by VITEK 2 (BioMérieux), Bruker MALDI Biotyper, and 16s rRNA gene sequencing. For determination of16S rRNA gene sequence, the almost complete coding region of 16S rRNA gene was amplified by PCR with the universal primers 27f (AGAGTTTGATCCTGGCTCAG) and 1492r (TACCTTGTTACGACTT)^30^ and then sequenced on ABI 3730 Sequencer.

### PCR detection of *bla* genes

All known carbapenemase and ESBL genes as listed in Table S1 were subjected to PCR detection. Primer pair GTCCAAGACCGGCAACTC/CACCAGCAGGATCGACAG was designed from whole-genome shotgun sequences of strain A22732 (data not shown) for amplifying the DNA fragments containing the whole coding region of *bla*_OXA-114_, because all the available primers gave negative amplification. All amplicons were sequenced on ABI 3730 Sequencer with the same primers for PCR.

### Detection of carbapenemase activity

Activity of class A/B/D carbapenemases was determined by CarbaNP test^6^ with modifications. Overnight bacterial cell culture in the Mueller-Hinton broth was diluted 1:100 into 3 ml of fresh Mueller-Hinton broth, and bacteria were allowed to grow at 37°C with shaking at 200 rpm to reach an optical density (OD_600_) of 1.0 to 1.4. If required, ampicillin was used at 100 μg/ml. Bacterial cells were harvested from 2 ml of the above culture, and washed twice with 20 mM Tris-HCl (pH 7.8). Cell pellets were resuspended in 500 μl of 20 mM Tris-HCl (pH 7.8), and lysed by soniation, followed by centrifugation at 10000 × g at 4°C for 5 min. 50 μl of the supernatant (the enzymatic bacterial suspension) were mixed with 50 μl of substrate I to V, respectively, followed by incubation at 37°C for a maximum of 2 h. Substrate I: 0.054% red phenol plus 0.1 mM ZnSO_4_ (pH7.8). Substrate II: 0.054% red phenol plus 0.1 mM ZnSO_4_ (pH7.8), and 0.6 mg/μl imipenem. Substrate III: 0.054% red phenol plus 0.1 mM ZnSO_4_ (pH7.8), 0.6 mg/μl mg imipenem, and 0.8 mg/μl tazobactam. Substrate IV: 0.054% red phenol plus 0.1 mM ZnSO_4_ (pH7.8), 0.6 mg/μl mg imipenem, and 3 mM EDTA (pH7.8). Substrate V: 0.054% red phenol plus 0.1 mM ZnSO_4_ (pH7.8), 0.6 mg/μl mg imipenem, 0.8 mg/μl tazobactam, and 3 mM EDTA (pH7.8).

### Conjugal transfer

Plasmid conjugal transfer experiments were carried out with rifampin-resistant *E. coli* EC600 being used as recipient and *bla*_IMP_-positive *A. xylosoxidans* as donor. 3 ml of overnight cultures of each of donor and recipient bacteria were mixed together, harvested and resuspended in 80 μl of Brain Heart Infusion (BHI) medium. The mixture was spotted on a 1 cm^2^ filter membrane that was placed on the BHI agar plate, and then incubated for mating at 37°C for 12-18 h. Bacteria were washed from the filter membrane and spotted on the Muller-Hinton agar plate containing 1500 μg/ml rifampin and 100 μg/ml ampicillin for selection of the *bla*_IMP_-positive *E. coli* transconjugant.

### Determination of minimum inhibitory concentration (MIC)

The MIC values of indicated bacterial strains were tested by using VITEK 2 according to manufacturer's instructions, and antimicrobial susceptibility was judged by Clinical and Laboratory Standards Institute (CLSI) standard.

### Determination of plasmid DNA sequence

The chromosome DNA-free plasmid DNA was isolated from the cell cultures of the *bla*_IMP_-positive *E. coli* transconjugant using a Qiagen large construct kit, and then sequenced by using whole-genome shotgun strategy in combination with Illumina HiSeq 2500 sequencing technology. The contigs were assembled with Velvet, and the gaps were filled through combinatorial PCR and Sanger Sequencing on ABI 3730 Sequencer. The genes were predicted with GeneMarkS and further annotated by BLASTP against Uniport and NR databases.

### RNA isolation and reverse transcription (RT)-PCR

Bacteria were cultured overnight in Mueller-Hinton broth (BD) with or without addition of 2 μg/ml imipenem (Sigma). Total RNAs were extracted from harvested bacterial cells using TRIzol Reagent (Life Technologies). RNA quality was monitored by agarose gel electrophoresis, and RNA quantity was determined by spectrophotometry. The contaminated DNA in the total RNA samples was removed by using Amibion's DNA-free^TM^ Kit. cDNAs were generated by using 5 µg of RNA and 3 µg of random hexamer primers in a 40 µl reaction mixture. The cDNA samples were generated by RT from total RNAs. Genomic DNA and cDNA were used as the templates for PCR and RT-PCR, respectively, with the primer pair TGTTTGATGTTATGGAGCAG/AGCCGTAAATGGAGTGTC. To ensure that no contamination of genomic DNA in the RT reactions would occur, RT-PCR of negative controls was performed using the ‘cDNA’ sample generated without reverse transcriptase as template. Reactions containing primer pairs without templates were also included as blank controls.

### Primer extension assay

The [γ-^32^P] ATP end-labeled primer CAGTCATAACAAGCCAT or CATACTTTTCCTTTTCTAACGG, which was complementary to *aacA7* or *bla*_IMP-1_ transcript, respectively, was annealed with total RNA sample of strain A22732 for primer extension assay as described previously^31^. For different cell cultures (lanes) in a single experiment, equal amounts of total RNA were used as starting materials. The corresponding end-labeled primer was also used for sequencing the PCR amplicon generated by the primer pair TGACGATGCGTGGAGACC/CAGTCATAACAAGCCAT or TGTTTGATGTTATGGAGCAG/AGCCGTAAATGGAGTGTC. DNA sequencing was carried out using the AccuPower & Top DNA Sequencing Kit (Bioneer). Primer extension products and sequencing materials were analyzed on an 8 M urea-6% polyacrylamide gel electrophoresis. Radioactive species were detected by autoradiography.

### Nucleotide sequence accession number

The complete sequence (File S1) of plasmid pA22732-IMP was submitted to the GenBank nucleotide sequence database under accession number KJ588780.

## Author Contributions

D.Z. and C.L. designed experiments. Z.C., H.F., L.W., F.S., Y.W., Z.Y., H.Y., W.Y., J.W., P.X., D.Z. and C.L. performed experiments. D.Z., Z.C. and H.F. analyzed data. D.Z., Z.C., H.F. and Y.W. contributed reagents, materials and analysis tools. D.Z. and C.L. wrote this manuscript.

## Supplementary Material

Supplementary Information Supplementary material

## Figures and Tables

**Table 1:** MIC values and antimicrobial susceptibility

	MIC/antimicrobial susceptibility		
Antibiotics	A22732	22732-IMP-EC600	EC600
Ampicillin	> = 32/R	> = 32/R	16//I
Ampicillin/sulbactam	> = 32/R	> = 32/R	< = 2/S
Aztreonam	> = 64/R	< = 1/S	< = 1/S
Cefazolin	> = 64/R	> = 64/R	< = 4/S
Cefuroxime sodium	> = 64/R	> = 64/R	16//I
Cefuroxime axetil	> = 64/R	> = 64/R	16//I
Cefotetan	> = 64/R	> = 64/R	< = 4/S
Ceftriaxone	> = 64/R	> = 64/R	< = 1/S
Ceftazidime	> = 64/R	> = 64/R	< = 1/S
Cefepime	> = 64/R	> = 64/R	< = 1/S
Imipenem	> = 16/R	4/R	< = 1/S
Meropenem	> = 16/R	2/R	< = 0.25/S
Ciprofloxacin	1/S	< = 0.25/S	< = 0.25/S
Levofloxacin	2/S	0.5/S	< = 0.25/S
Macrodantin	256/R	< = 16/S	< = 16/S
Amikacin	> = 64/R	< = 2/S	< = 2/S
Gentamicin	> = 16/R	< = 1/S	< = 1/S
Tobramycin	> = 16/R	4/S	< = 1/S
Trimethoprim/sulfamethoxazole	< = 20/S	< = 20/S	< = 20/S

S = sensitive; R = resistant; I = Intermediate.
