# Supplementary material for: IMP-1 encoded by a novel Tn402-like class 1 integron in clinical Achromobacter xylosoxidans, China
Source: Sci Rep. 2014 Nov 27;4:7212. doi: 10.1038/srep07212 (PMC4245530; doi:10.1038/srep07212)
Supplement: Supplementary Information — Supplementary material [file srep07212-s1.doc]

**IMP-1 encoded by a novel Tn*402*-like class 1 integron in clinical *Achromobacter xylosoxidans*, China**

Zhenhong Chen, Haihong Fang, Li Wang, Fengjun Sun, Yong Wang, Zhe Yin, Huiying Yang, Wenhui Yang, Jie Wang, Peiyuan Xia, Dongsheng Zhou, Changting Liu

**Table S1 PCR detection of carbapenemase and extended-spectrum ß-lactamase genes**

| **ß-lactamase** | | | | **PCR detection** | | | |
| --- | --- | --- | --- | --- | --- | --- | --- |
| **Ambler**  **class** | **Type** | **Subtype** | **Gene** | **Primers** | **AL** | **AT** | **Reference** |
| **Carbapenemases** | | | | | | | |
| A | GES | GES-2, GES-4 to GES-6, GES-11, GES-14, GES-18 | *bla*_GES_ | GES-F: GCTTCATTCACGCACTATT  GES-R: CGATGCTAGAAACCGCTC | 323 | 52 |  |
|  | KPC | KPC-1 to KPC-15 | *bla*_KPC_ | KPC-F: GTATCGCCGTCTAGTTCTGC  KPC-R: GGTCGTGTTTCCCTTTAGCC | 638 | 56 |  |
|  | SME | SME-1 to SME-3 | *bla*_SME_ | SME-F1: GAGGAAGACTTTGATGGGAGGAT  SME-R1: TCCCCTCAGGACCGCCAAG | 334 | 52 |  |
|  | IMI  (NMC-A) | IMI-1 to IMI-3 | *bla*_IMI_ | IMI-F: TGCGGTCGATTGGAGATAAA  IMI-R: CGATTCTTGAAGCTTCTGCG | 399 | 52 |  |
|  | BIC | BIC-1 | *bla*_BIC_ | BIC-F: TATGCAGCTCCTTTAAGGGC  BIC-R: TCATTGGCGGTGCCGTACAC | 537 | 52 |  |
| B1 | IMP | IMP-1 to IMP-44 | *bla*_IMP_ | IMP-F: GGAATAGAGTGGCTTAAYTCTC  IMP-R: GGTTTAAYAAAACAACCACC | 232 | 56 |  |
|  | VIM | VIM-1 to VIM-37 | *bla*_VIM_ | VIM-F: GATGGTGTTTGGTCGCATA  VIM-R: CGAATGCGCAGCACCAG | 390 | 52 |  |
|  | NDM | NDM-1 to NDM-8 | *bla*_NDM_ | NDM-F: GGTTTGGCGATCTGGTTTTC  NDM-R: CGGAATGGCTCATCACGATC | 621 | 56 |  |
|  | TMB | TMB-1 to TMB-2 | *bla*_TMB_ | TMB-F: CAAGGAGCTCATTCAAAGG  TMB-R: TTCTAGCGGATTGTGGCCAC |  | 52 |  |
|  | FIM | FIM-1 | *bla*_FIM_ | FIM-F: GAAGCACATGGAAAACTGGG  FIM-R: GATGGGCGAATGAGACAGC |  | 52 |  |
|  | SPM | SPM-1 | *bla*_SPM_ | SPM-F: AAAATCTGGGTACGCAAACG  SPM-R: ACATTATCCGCTGGAACAGG | 271 | 52 |  |
|  | DIM | DIM-1 | *bla*_DIM_ | DIM-F: GCTTGTCTTCGCTTGCTAACG  DIM-R: CGTTCGGCTGGATTGATTTG | 699 | 52 |  |
|  | GIM | GIM-1 | *bla*_GIM_ | GIM-F: TCGACACACCTTGGTCTGAA  GIM-R: AACTTCCAACTTTGCCATGC | 477 | 52 |  |
|  | SIM | SIM-1 | *bla*_SIM_ | SIM-F: TACAAGGGATTCGGCATCG  SIM-R: TAATGGCCTGTTCCCATGTG | 570 | 52 |  |
| B3 | AIM | AIM-1 | *bla*_AIM_ | AIM-F: CTGAAGGTGTACGGAAACAC  AIM-R: GTTCGGCCACCTCGAATTG | 322 | 52 |  |
|  | SMB | SMB-1 | *bla*_SMB_ | SMB-F: CAGCAGCCATTCACCATCTA  SMB-R: GAAGACCACGTCCTTGCACT | 492 | 52 |  |
| D | OXA | OXA-23-like | *bla*_OXA-23-like_ | OXA-23-F: GATCGGATTGGAGAACCAGA  OXA-23-R: ATTTCTGACCGCATTTCCAT | 501 | 56 |  |
|  |  | OXA-24-like | *bla*_OXA-24-like_ | OXA-24-F: GGTTAGTTGGCCCCCTTAAA  OXA-24-R: AGTTGAGCGAAAAGGGGATT | 246 | 52 |  |
|  |  | OXA-48-like | *bla*_OXA-48-like_ | OXA-48-F: TTGGTGGCATCGATTATCGG  OXA-48-R: GAGCACTTCTTTTGTGATGGC | 744 | 52 |  |
|  |  | OXA-58-like | *bla*_OXA-58-like_ | OXA-58-F: AAGTATTGGGGCTTGTGCTG  OXA-58-R: CCCCTCTGCGCTCTACATAC | 599 | 56 |  |
|  |  | OXA-143-like | *bla*_OXA-143-like_ | OXA-143-F: TGGCACTTTCAGCAGTTCCT  OXA-143-R: TAATCTTGAGGGGGCCAACC | 149 | 52 |  |
|  |  | OXA-235-like | *bla*_OXA-235-like_ | OXA-235-F: TTGTTGCCTTTACTTAGTTGC  OXA-235-R: CAAAATTTTAAGACGGATCG | 768 | 52 |  |
|  |  | OXA-114 | *bla*_OXA-114_ | OXA-114-F: CGCATCCTGTTCCAGCA  OXA-114-R: GTGCCGGTCTTGCCATAC | 509 | 52 |  |
| **Extended-spectrum ß-lactamases** | | | | | | | |
| A | CTX-M | CTX-M universal | *bla*_CTX-M universal_ | CTX-M-UF: ATGTGCAGYACCAGTAARGT  CTX-M-UR: TGGGTRAARTARGTSACCAGA | 593 | 52 |  |
|  |  | CTX-M-1 group | *bla*_CTX-M-1 group_ | CTX-M-1GF: AAAAATCACTGCGCCAGTTC  CTX-M-1GR: AGCTTATTCATCGCCACGTT | 415 | 52 |  |
|  |  | CTX-M-2 group | *bla*_CTX-M-2 group_ | CTX-M-2GF: CGACGCTACCCCTGCTATT  CTX-M-2GR: CCAGCGTCAGATTTTTCAGG | 552 | 52 |  |
|  |  | CTX-M-8 group | *bla*_CTX-M-8 group_ | CTX-M-8GF: TCGCGTTAAGCGGATGATGC  CTX-M-8GR: AACCCACGATGTGGGTAGC | 666 | 52 |  |
|  |  | CTX-M-9 group | *bla*_CTX-M-9 group_ | CTX-M-9GF: CAAAGAGAGTGCAACGGATG  CTX-M-9GR: ATTGGAAAGCGTTCATCACC | 205 | 52 |  |
|  |  | CTX-M-25 group | *bla*_CTX-M-25 group_ | CTX-M-25GF: GCACGATGACATTCGGG  CTX-M-25GR: AACCCACGATGTGGGTAGC | 327 | 52 |  |
|  | TEM |  | *bla*_TEM_ | TEM-F: CATTTCCGTGTCGCCCTTATTC  TEM-R: CGTTCATCCATAGTTGCCTGAC | 800 | 52 |  |
|  | SHV |  | *bla*_SHV_ | SHV-F: AGCCGCTTGAGCAAATTAAAC  SHV-R: ATCCCGCAGATAAATCACCAC | 713 | 52 |  |
|  | GES |  | *bla*_GES_ | GES-ESBL-F: AGTCGGCTAGACCGGAAAG  GES-ESBL-R: TTTGTCCGTGCTCAGGAT | 399 | 52 |  |
|  | PER |  | *bla*_PER_ | PER-F: GCTCCGATAATGAAAGCGT  PER-R: TTCGGCTTGACTCGGCTGA | 520 | 52 |  |
|  | VEB |  | *bla*_VEB_ | VEB-F: CATTTCCCGATGCAAAGCGT VEB-R: CGAAGTTTCTTTGGACTCTG | 648 | 52 |  |
| D | OXA | OXA-1 group | *bla*_OXA-1 group_ | OXA-1-F: GGCACCAGATTCAACTTTCAAG  OXA-1-R: GACCCCAAGTTTCCTGTAAGTG | 564 | 52 |  |

AL: amplicon length (bp); AT: annealing temperature (°C)

**Fig S1 Detection of carbapenemase activity**

**
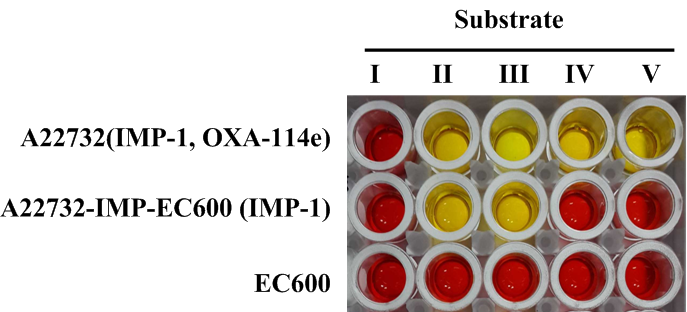
**

In the presence of any carbapenemase, relevant carbapenems are hydrolyzed and transformed into its carboxylic form, thus leading to a pH decrease which is detected by a color change of phenol red solution (red to yellow-orange). Ambler class A carbapenemases are, at least partially, inhibited by tazobactam, whereas class B carbapenemases (metallo-ß-lactamases) are inhibited by divalent cation chelators such as EDTA. There is no available chemical inhibitor for class D carbapenemases. In this study, 22732-IMP-EC600 had class B carbapenemase activity, while A22732 probably expressed class B/D carbapenemases, being consistent with PCR/sequencing results of antibiotics resistance genes (see main text). As expected, *E. coli* EC600 had no carbapenemase activity.

**Fig S2 Evolution of Tn402-like class 1 integrons**

**
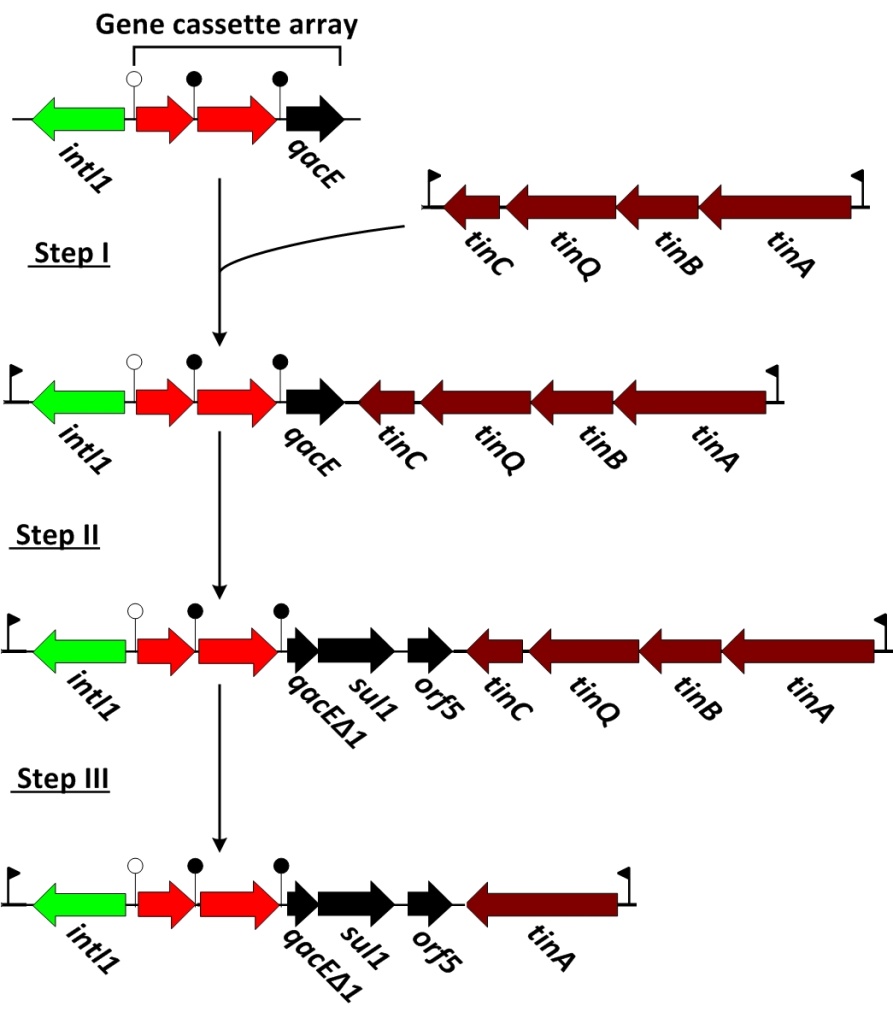
**

Genes are denoted by arrows and colored based on gene function classification. At least three key steps are involved in evolution of Tn*402*-like class 1 integrons : step I, insertion of ancestor class 1 integron (lack of 3′- CS) into Tn*402* (harboring complete *tniABQC* transposition module) to generate a hybrid structure, combining the ability of integron to capture environmental gene cassettes to the mobility of Tn*402* into plasmids and other genetic platforms, which might occur prior to or concomitant with antibiotic era including capture of *qacE* (quaternary ammonium compound resistance); step II, capture of *sul1* (sulfonamide resistance) and *orf5*, and then formation of 3’-CS due to deletion events between *qacE* and *sul1*; step III, deletion events within *tniABQR* , making Tn*402* transposition incompetent.

**References**

1. Hong SS, Kim K, Huh JY, Jung B, Kang MS, Hong SG: Multiplex PCR for rapid detection of genes encoding class A carbapenemases*.* *Annals of laboratory medicine* 32(5), 359-361 (2012).

2. Poirel L, Walsh TR, Cuvillier V, Nordmann P: Multiplex PCR for detection of acquired carbapenemase genes*.* *Diagn Microbiol Infect Dis* 70(1), 119-123 (2011).

3. El Salabi A, Borra PS, Toleman MA, Samuelsen O, Walsh TR: Genetic and biochemical characterization of a novel metallo-beta-lactamase, TMB-1, from an Achromobacter xylosoxidans strain isolated in Tripoli, Libya*.* *Antimicrob Agents Chemother* 56(5), 2241-2245 (2012).

4. Pollini S, Maradei S, Pecile P *et al.*: FIM-1, a new acquired metallo-beta-lactamase from a Pseudomonas aeruginosa clinical isolate from Italy*.* *Antimicrob Agents Chemother* 57(1), 410-416 (2013).

5. Wachino J, Yoshida H, Yamane K *et al.*: SMB-1, a novel subclass B3 metallo-beta-lactamase, associated with ISCR1 and a class 1 integron, from a carbapenem-resistant Serratia marcescens clinical isolate*.* *Antimicrob Agents Chemother* 55(11), 5143-5149 (2011).

6. Woodford N, Ellington MJ, Coelho JM *et al.*: Multiplex PCR for genes encoding prevalent OXA carbapenemases in Acinetobacter spp*.* *Int J Antimicrob Agents* 27(4), 351-353 (2006).

7. Poirel L, Potron A, Nordmann P: OXA-48-like carbapenemases: the phantom menace*.* *J Antimicrob Chemother* 67(7), 1597-1606 (2012).

8. Higgins PG, Lehmann M, Seifert H: Inclusion of OXA-143 primers in a multiplex polymerase chain reaction (PCR) for genes encoding prevalent OXA carbapenemases in Acinetobacter spp*.* *Int J Antimicrob Agents* 35(3), 305 (2010).

9. Higgins PG, Perez-Llarena FJ, Zander E, Fernandez A, Bou G, Seifert H: OXA-235, a novel Class D Beta-Lactamase Involved in Resistance to Carbapenems in Acinetobacter baumannii*.* *Antimicrob Agents Chemother*, (2013).

10. Turton JF, Mustafa N, Shah J, Hampton CV, Pike R, Kenna DT: Identification of Achromobacter xylosoxidans by detection of the bla(OXA-114-like) gene intrinsic in this species*.* *Diagn Microbiol Infect Dis* 70(3), 408-411 (2011).

11. Pagani L, Dell'amico E, Migliavacca R *et al.*: Multiple CTX-M-type extended-spectrum beta-lactamases in nosocomial isolates of Enterobacteriaceae from a hospital in northern Italy*.* *J Clin Microbiol* 41(9), 4264-4269 (2003).

12. Woodford N, Fagan EJ, Ellington MJ: Multiplex PCR for rapid detection of genes encoding CTX-M extended-spectrum (beta)-lactamases*.* *J Antimicrob Chemother* 57(1), 154-155 (2006).

13. Dallenne C, Da Costa A, Decre D, Favier C, Arlet G: Development of a set of multiplex PCR assays for the detection of genes encoding important beta-lactamases in Enterobacteriaceae*.* *J Antimicrob Chemother* 65(3), 490-495 (2010).

14. Sajjad A, Holley MP, Labbate M, Stokes HW, Gillings MR: Preclinical class 1 integron with a complete Tn402-like transposition module*.* *Appl Environ Microbiol* 77(1), 335-337 (2011).

15. Gillings M, Boucher Y, Labbate M *et al.*: The evolution of class 1 integrons and the rise of antibiotic resistance*.* *J Bacteriol* 190(14), 5095-5100 (2008).

16. Toleman MA, Vinodh H, Sekar U, Kamat V, Walsh TR: blaVIM-2-harboring integrons isolated in India, Russia, and the United States arise from an ancestral class 1 integron predating the formation of the 3' conserved sequence*.* *Antimicrob Agents Chemother* 51(7), 2636-2638 (2007).

17. Stokes HW, Nesbo CL, Holley M, Bahl MI, Gillings MR, Boucher Y: Class 1 integrons potentially predating the association with tn402-like transposition genes are present in a sediment microbial community*.* *J Bacteriol* 188(16), 5722-5730 (2006).
